# Cholesterol: An important actor on the cancer immune scene

**DOI:** 10.3389/fimmu.2022.1057546

**Published:** 2022-11-21

**Authors:** Hossein Halimi, Shirin Farjadian

**Affiliations:** Department of Immunology, School of Medicine, Shiraz University of Medical Sciences, Shiraz, Iran

**Keywords:** cholesterol, immune cells, cancer, anticholesteremic agents, immunometabolism

## Abstract

Based on the structural and signaling roles of cholesterol, which are necessary for immune cell activity, high concentrations of cholesterol and its metabolites not only trigger malignant cell activities but also impede immune responses against cancer cells. To proliferate and evade immune responses, tumor cells overcome environmental restrictions by changing their metabolic and signaling pathways. Overexpression of mevalonate pathway enzymes and low-density lipoprotein receptor cause elevated cholesterol synthesis and uptake, respectively. Accordingly, cholesterol can be considered as both a cause and an effect of cancer. Variations in the effects of blood cholesterol levels on the outcome of different types of cancer may depend on the stage of cancer. However, positive effects of cholesterol-lowering drugs have been reported in the treatment of patients with some malignancies.

## Introduction

In addition to the physical role of fatty acids, they play multifaceted roles in cancer. Depending on their composition, lipids may have inflammatory or anti-inflammatory impacts on the tumor microenvironment (1, 2).

The functions of cholesterol in cancer were first noted in the 1900s. Webb reported that cholesterol crystallization in normal cells led to malignancy (3), and other researchers subsequently found alterations in blood cholesterol levels in patients with cancer (4, 5). Recently, cholesterol was considered not only a risk factor, but also a prognostic factor in cancer (6), as high-density lipoprotein (HDL) cholesterol was reported to have impact on patient survival and cancer relapse (7). Indeed, cholesterol accumulation has been observed in different tumor cells (8, 9), through the upregulation of cholesterol biosynthesis or *via* increased uptake. Many of cancer cells overexpress low-density lipoprotein receptor (LDLR) compared to normal cells, and evade negative feedback mechanisms for enhancing cholesterol uptake – a phenomenon which leads to their rapid proliferation (10–13).

Cholesterol is crucial to triggering immune responses as one of the components of the lipid raft, which can recruit receptors and signaling molecules (14). Proteins are also localized in the lipid raft *via* the palmitoylation or glycosylphosphatidylinositol (GPI) anchor (15). As a result of cholesterol accumulation, tumor cells have higher membrane levels of cholesterol, and this structure plays a role in tumor cell growth, adhesion, migration, invasion and apoptosis (16).

Indeed, Cholesterol metabolism is associated with cancer stemness. Cancer stem cells are a population within cancer cells related to progression and metastasis (17). Ehmsen et al. demonstrated that raised *de novo* cholesterol synthesis is a feature of breast cancer stem cells and inhibiting cholesterol synthesis impedes the growth of cancer stem cells (18).

Cholesterol is a signaling molecule that in turn affects other signaling pathways such as PI3K and Hedgehog (19). The metabolism of cholesterol impacts the cancer-associated immune system through altering responses such as the induction of exhausted T cells, changing macrophage phenotype, and affecting antigen presentation by dendritic cells (DCs) (20).

Targeting cholesterol biosynthesis or uptake is a new area in cancer therapy. In efforts to regulate blood cholesterol levels as well as research in other treatment modalities such as immunotherapy, further insights into cholesterol biosynthesis and uptake may be applied in strategies to prevent tumor relapse or metastasis, and may extend patient survival (21).

## Cholesterol synthesis

Although most human cells are able to synthesize cholesterol, the liver is the leading site of *de novo* cholesterol synthesis. This pathway comprises of more than 20 enzymes (22), and starts with the conversion of two acetyl-CoA by thiolase and then further conversion of acetyl-CoA into 3-hydroxy-3-methylglutaryl (HMG)-CoA. This is followed by the production of other intermediates such as isopentyl pyrophosphate, farnesyl pyrophosphate, squalene, lanosterol, and finally cholesterol (20). Elucidating the cholesterol synthesis pathway is important because mediators and intermediate enzymes affect the cancer process. For example, C-terminal farnesylation of proteins leads to the formation of a hydrophobic tail, thus enabling lipid–protein and protein–protein interaction (23–25). Indeed, farnesylation is a necessary step for RAS oncoprotein activation and cell membrane binding. Moreover, inhibition of this process was shown to have anti-tumor effects (26, 27). Thus, current evidence suggests that deeper knowledge of the cholesterol synthesis pathway can point the way to novel cancer treatments and the potential to extend patients’ lifespans.

## Cancer-associated cholesterol metabolism

**Figure 1:**
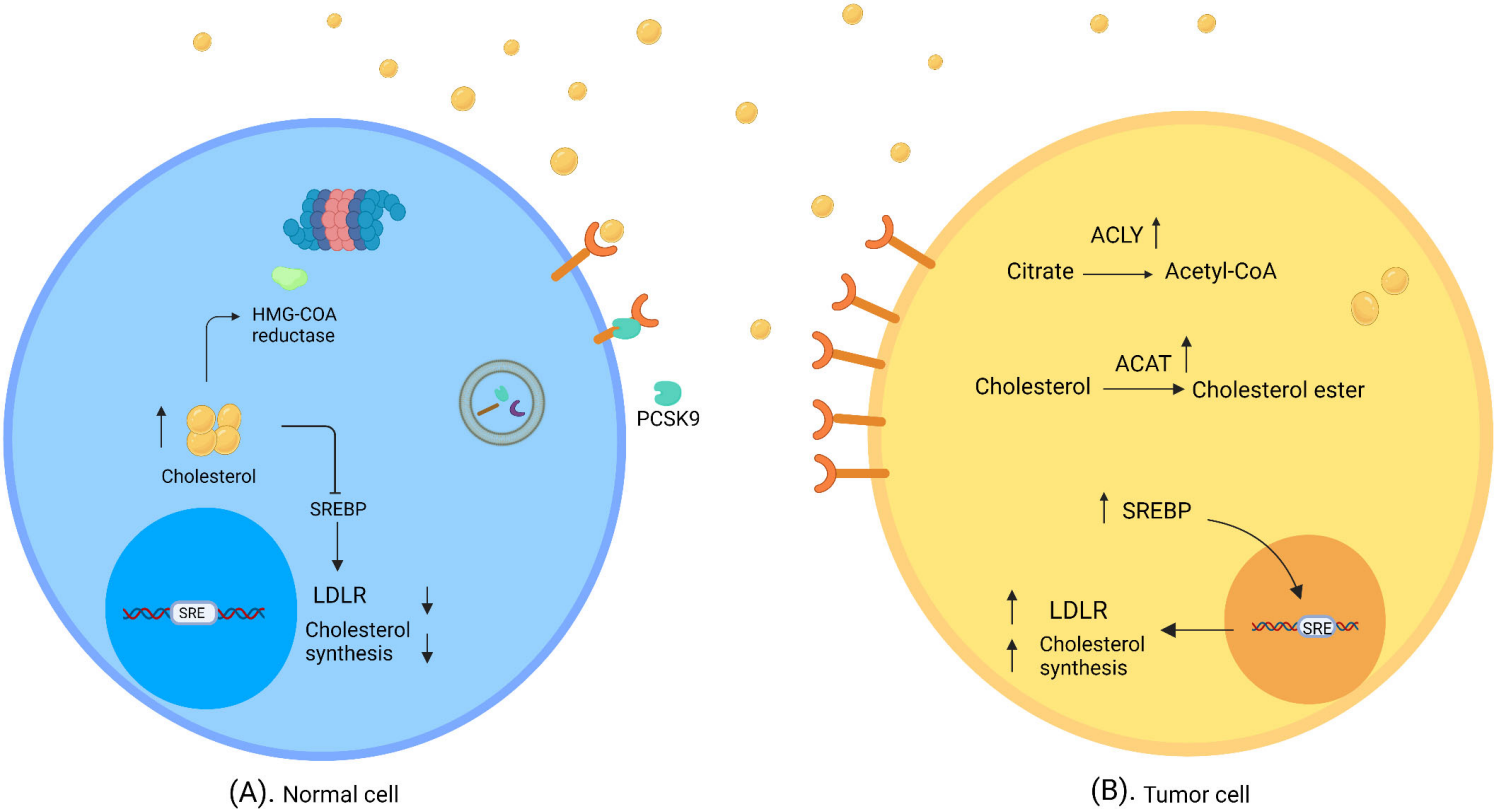
Normal and tumor cells in cholesterol-rich environment. **(A)** In normal cells, a high concentration of cholesterol triggers regulatory mechanisms of cholesterol synthesis and uptake, which downregulate SREBP and degrade HMG- CoA reductase. **(B)** Tumor cells evade feedback mechanisms in a cholesterol-rich environment. SREBP shows high activity; thus LDLR expression and/or cholesterol synthesis are elevated. ACAT catalyzes the synthesis of cholesterol ester; therefore, cholesterol accumulates in tumor cells. ATP citrate lyase converts citrate to acetyl-CoA and induces fatty acid synthesis (created with biorender.com).

Cholesterol concentrations within the cell are precisely controlled through the regulation of absorption and synthesis (**Figure 1**). Cholesterol absorption is firmly controlled by regulating the amount of its receptor. High levels of cholesterol prevent the activation of SREBPs (sterol regulatory-element binding proteins), i.e. membrane-bound transcription factors that activate genes involved in cholesterol synthesis, thereby reducing LDLR expression (28, 29). In addition, PCSK9 (proprotein convertase subtilisin/kexin type 9) binds to LDLR and thus triggers its breakdown (30). Cholesterol synthesis is also tightly controlled, as high cholesterol levels cause the degradation of squalene monooxygenase and HMG-CoA reductase, which catalyzes the conversion of squalene to 2,3 oxidosqualene and HMG-CoA to mevalonate as the precursors of cholesterol (31, 32).

To utilize more cholesterol, cancer cells evade the feedback mechanisms of cholesterol inhibition. In this connection it was found that prostate cancer cells overexpress LDLR and SREBP in cholesterol-rich conditions in the absence of cholesterol regulatory mechanisms (10).

Cholesterol absorption is more energy-saving in cancer cells than *de novo* synthesis. Accordingly, in large-cell lymphoma, malignant cells rely more on cholesterol uptake through the overexpression of LDLR rather than *de novo* synthesis (33). This might be explained by the higher LDLR expression in inflammatory conditions, which enables tumor cells to accumulate more cholesterol (34). In addition, prostate cancer cells were reported to accumulate excess cholesterol from the environment through the overexpression of HDLR, SR-B1 (35); however, advanced-stage prostate cancer cells were reported to be dependent on cholesterol synthesis (36).

Another factor that supports cancer progression through cholesterol synthesis is ATP citrate lyase (ACLY) enzyme, which converts citrate to acetyl-CoA. Overexpression of this enzyme has been reported in different kinds of tumor (37).

Because of insufficient vascularization and a high rate of growth, some cancers such as glioblastoma exist in a hypoxic and lipid-limited microenvironment. In this condition, SREBP2 overexpression induces cholesterol synthesis (38, 39).

Cholesterol ester is a storage form of cholesterol catalyzed by acyl-CoA: cholesterol acyltransferase (ACAT), which plays an important role in cancer biology. Cholesterol acyltransferase overexpression has been detected in cancer tissues, and it appears that cancer cells use this storage source for their proliferation (40). In addition, lysosomal acid lipase breaks down cholesterol ester to generate fatty acids; the overexpression of this enzyme and its relation to cancer progression have been reported in renal cell carcinoma (41).

Oxysterols, i.e. oxygenated sterol derivatives such as different forms of hydroxycholesterols, are bioactive molecules which affect cancer cell behavior (42). A role for 27-hydroxycholesterol has been shown in breast cancer progression through VEGF overexpression and STAT3 activation (43).

## Cholesterol and cancer cell signaling

### Hedgehog pathway

The Hedgehog signaling pathway is an essential process which enables appropriate cell differentiation during vertebrate embryonic development. It is also necessarily activated in circumstances such as wound healing and tissue repair. It has been shown that this pathway is involved in cancer growth, and that cholesterol plays a crucial role in the reactivation of this pathway through binding to smoothened transmembrane protein (44–46).

### Wnt pathway

The Wnt pathway is responsible for stem cell renewal and organogenesis. This pathway is also involved in the development of malignancies such as leukemia and gastrointestinal cancer. The Wnt protein interacts with a heterodimeric receptor including Frizzled and LRP5/6 proteins. Dishevelled and other signaling molecules are then recruited to the receptor and transduce the signal through activated β-catenin (47, 48). It has been indicated that cholesterol can bind to the PDZ domain of scaffold proteins including NHERF1/EBP50, and then activate Wnt signaling pathways (49, 50). An additional way in which cholesterol plays a role in cancer biology is by triggering the Wnt pathway through its interaction with Dishevelled scaffold protein (51).

### mTORC1 pathway

The mTORC1 pathway is a key factor in cell growth and survival. It is also suggested that mTORC1 is involved in cancer cell proliferation. This signaling pathway is responsible for lipid synthesis in malignant cells *via* SREBP2 upregulation (52, 53).

Cholesterol can increase tumor cell proliferation through activation of the mTORC1 pathway at the lysosomal surface by SLC38A9 (solute carrier family 38 member 9), a lysosomal transmembrane protein that senses cholesterol in addition to arginine, with independent motifs (54).

## Relevance of cholesterol and cancer-associated immune cell

**Figure 2:**
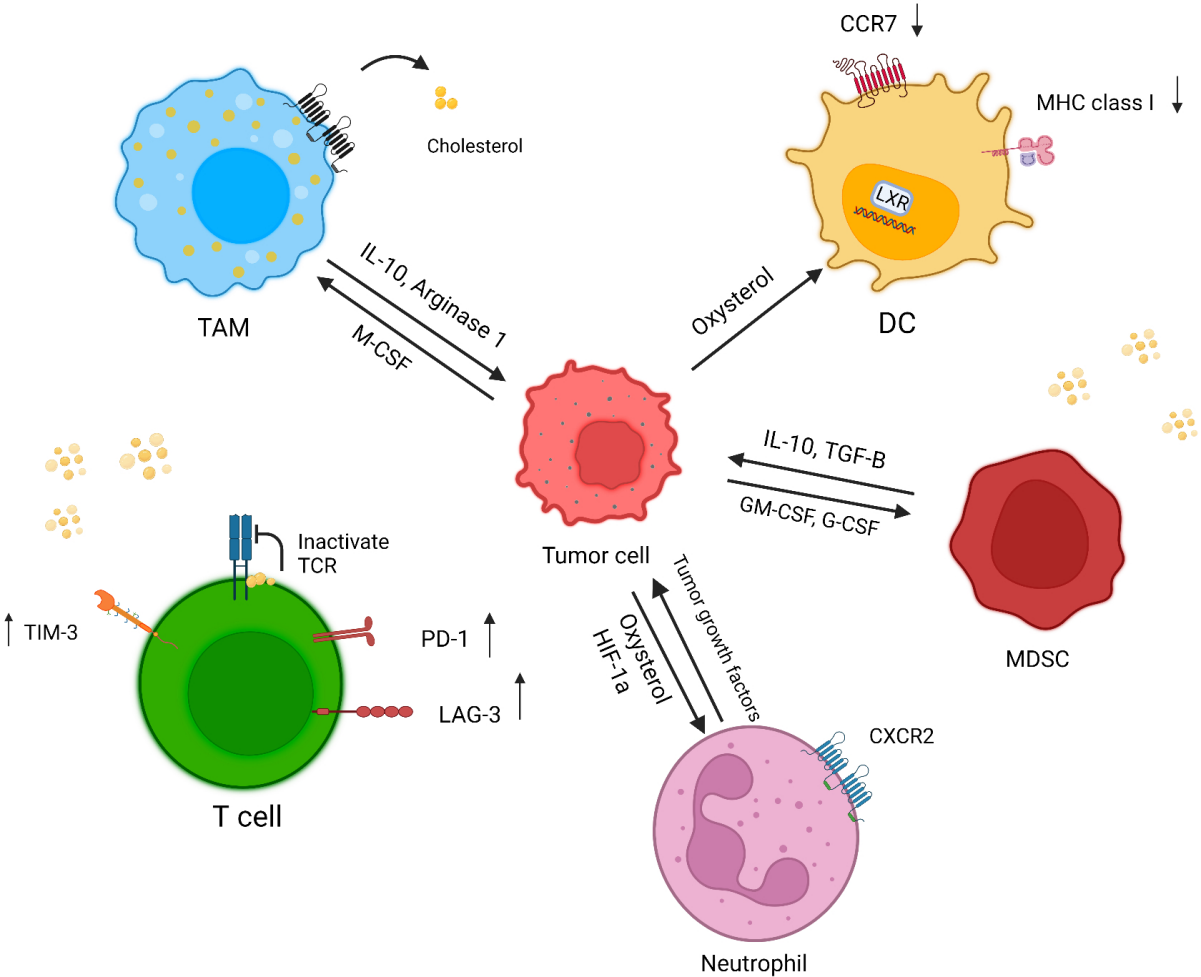
Effect of a cholesterol-rich tumor microenvironment on immune cells. Increased cholesterol efflux from macrophage membranes induces the M2 phenotype in tumor resident macrophages. In cholesterol-rich area, T cells overexpress exhaustion markers, and cholesterol binds to the TCR β-chain and puts T cells in a resting state. Oxysterol recruits CXCR2-positive neutrophils, which in turn induces angiogenesis and tumor growth. Tumor-released factors induce MDSC suppressive responses through cholesterol accumulation. The accumulation of cholesterol in DCs causes a reduction in MHC class I and CCR7 expression, which diminishes the immune response against tumor cells (created with biorender.com).

Although cholesterol is essential for the formation of the immunological synapse through receptor clustering and recruitment of signaling molecules, high amounts cholesterol have a negative effect on immune responses (55) (**Figure 2**).

### Macrophages

Macrophages are innate immune cells with a dual effect on cancer immunity. These cells show plasticity depending on the tumor microenvironment. At an advanced stage of cancer, macrophages convert to the M2 phenotype with tumor-promoting function (56).

Cholesterol has a pivotal effect on macrophage phenotype and function. Goossens et al. showed increased cholesterol efflux from the macrophage membrane and depletion of cholesterol from the lipid raft induced the tumor-promoting phenotype in tumor resident macrophages in a mouse model of ovarian cancer (57). Park et al. also revealed tumor-derived M-CSF activated PPARβ/δ (peroxisome proliferator-activated receptor belonging to the nuclear hormone receptor family) through increased fatty acid synthesis, thereby augmenting IL-10 and arginase 1 expression in macrophages, which resulted in cancer invasion and angiogenesis (58).

### Dendritic cells

MHC molecules play a pivotal role in tumor antigen presentation to immune cells and the induction of anti-tumor responses (59, 60). Dendritic cells are innate immune cells with a central role in triggering an anti-tumor response by presenting tumor antigens to T cells. Cancer cells can downmodulate the function of DCs by increasing the concentration of cholesterol (61).

Oxysterol secretion by tumor cells affects LXRs (liver x receptors) in DCs, which leads to CCR7 reduction, hampering the migration of DCs to lymph nodes and thus reducing T cell priming (62, 63). In addition, a high amount of lipid in the tumor microenvironment increases the uptake and accumulation of lipids in DCs, which in turn reduces tumor antigen presentation by these cells (64). It is also showed that oxidized lipids reduce the expression of MHC class I on DCs (65).

### T lymphocytes

Cholesterol has a dual effect on T cell function. During T cell responses, SREBPs exert an effect on cell proliferation through cholesterol synthesis (66). The results of one earlier study showed that cholesterol can bind to the transmembrane part of the TCR β-chain and keep T cells in the resting state (67). In addition, the tumor microenvironment is a cholesterol-rich milieu which leads to endoplasmic reticulum stress; as a result, T cells express more inhibitory receptors such as Tim-3, LAG3 and PD-1, and become exhausted. On the other hand cholesterol-lowering drugs such as atorvastatin were reported to decrease checkpoint inhibitors on T cells (68, 69). Although both CTLs and target cells are exposed to perforin within the synapse, high cholesterol content in the CTL cell membrane protects them against lysis by perforin (70).

In conclusion, although a sufficient concentration of cholesterol is needed for an efficient immune response, high levels of cholesterol can disrupt immune cell functioning (55).

### Neutrophils

There is cross-talk between cancer cells and neutrophils, the latter of which can play an anti-tumor or tumor-promoting role. In this connection, it was shown that microenvironmental conditions such as lipid level can change neutrophil functions (71).

Tumor-secreted oxysterols recruit CXCR2^+^ neutrophils that can release tumor growth factors (72, 73). In addition, hypoxia-induced Hif1 can elevate the expression of CYP46A1 (cytochrome P450 family 46 subfamily A member 1). This enzyme plays a role in cholesterol metabolism in the brain, and converts cholesterol to 24s-HC oxysterol, which can pass through the blood–brain barrier. One study showed that lipid accumulation recruited proangiogenic neutrophils in a pancreatic neuroendocrine tumor model (74).

### Myeloid-derived suppressor cells

Myeloid-derived suppressor cells (MDSCs) originate from immature or myeloid progenitor cells, and potentiate suppressive responses. Hence these cells can support cancer growth through regulatory mechanisms such as interference with T cell trafficking, release of suppressive factors (IL-10 and TGF-β), and depletion of essential T cell amino acids. Furthermore, tumor cells can induce MDSC responses through lipid-mediated mechanisms (75).

MDSCs are heterogeneous cells which are divided into two major groups: polymorphonuclear MDSCs (PMN-MDSCs) and monocytic MDSCs (M-MDSCs). Lectin-type oxidized LDL receptor 1 (Lox-a1) has been identified as one of the newest PMN-MDSC markers, which is evidence of a probable suppressive effect of cholesterol on these cells (76). Moreover, tumor-secreted G-CSF and GM-CSF induce the expression of lipid transporters on MDSCs through STAT3 and STAT5 signaling; consequently, elevated lipid absorption and accumulation trigger a suppressive response of MDSCs (77), **Table 1**.

**Table 1:** Effects of high cholesterol levels on cancer cells and immune cells.

Cancer cells	Immune cells
Overexpression of VEGF and angiogenesis	Reduce CCR7 and MHC class I on DCs
ROS generation	T cells exhaustion
Stemness and proliferation	Recruitment of CXCR2 positive neutrophils
Metastasis	Induce tumor-promoting macrophages
Drug resistance	Elevate MDSC response

## The effect of cholesterol on different types of cancer

Hypercholesterolemia is considered a risk factor for the induction of different types of cancer. Moreover, high cholesterol levels have also been shown to play a role in cancer progression and metastasis.

### Colon cancer

Blood concentrations of cholesterol in patients with colon cancer were reported to be lower than in healthy individuals (78, 79). The higher expression of LDLR by colon cancer cells results in decreased blood cholesterol levels, and colon cancer surgery can elevate cholesterol levels after 1 year (11).

LDL increases intestinal inflammation and the proliferation of malignant colon cells through the generation of reactive oxygen species (ROS) and activation of the MAPK signaling pathway. In addition, LDL promotes malignant colon cell migration and induces stemness genes such as *Sox2* and *Oct4* in these cells (13).

### Breast cancer

There is controversy regarding the relevance of cholesterol levels in breast cancer, although cholesterol is generally considered a risk factor. A recent report noted increased body mass index (BMI) as an important factor associated with breast cancer (80). Moreover, a cholesterol-rich diet reportedly elevates the risk of breast cancer. In this study, they found that high levels of cholesterol in patients with breast cancer are related to a poor outcome and a higher malignant cell proliferation rate (81).

In this connection, abundant expression of LDLR was seen in breast cancer cells (82), and LDL was reported to have an impact on breast cancer cell proliferation in association with overexpression of Akt and ERK pathway intermediates (83). Moreover, overexpression of CYP27A1 (sterol 27-hydroxylase), which produces 27-hydroxycholesterol, was reported in patients with breast cancer. This oxysterol downregulates the expression of P53,which in turn results in epithelial–mesenchymal transition and initiates the metastatic cascade (84, 85).

### Prostate cancer

There is controversy regarding the correlation between blood cholesterol levels and prostate cancer (86–88). High cholesterol synthesis and elevated LDLR expression may play a role in the outcome of this cancer (36); in this connection, high LDLR expression has been demonstrated in prostate cancer. Also, elevated amounts of cholesterol and cyclin E overexpression were detected inside of nucleus; hence cholesterol may play a role in the cell cycle of prostate cancer cells (89). In addition, androgen is necessary for prostate cancer cell proliferation, and prostate cancer cells were reported to gradually gain androgen synthesis potential by themselves in cholesterol-rich conditions (90, 91).

### Hepatocellular cancer

The liver is the primary source of cholesterol synthesis in the body (92). Many studies have documented high cholesterol concentrations in patients with hepatocellular cancer. It has been suggested that malignant cells are responsible for elevated cholesterol levels in these patients (93, 94). In this connection, the impairment of cholesterol feedback mechanisms was shown to have a substantial impact on lipid production in malignant liver cells (95).

### Lung cancer

It is currently hard to delineate specific correlations between lung cancer and blood cholesterol levels. The results of some studies showed that patients with low cholesterol levels have lower survival (96, 97). In contrast, one report suggested that elevated cholesterol is a risk factor for lung cancer (98). In this connection, a recent study suggested that moderate cholesterol levels can prevent lung cancer, whereas high and low cholesterol concentrations are both risk factors for this malignancy (99). The results of an *in vitro* study showed that 25-hydroxycholesterol promoted lung adenocarcinoma cell migration and invasion (100). It remains to be seen whether or not elevated cholesterol is a hallmark of lung cancer.

### Pancreatic cancer

The association between blood cholesterol concentration and pancreatic cancer remains unclear. Although one study found high cholesterol intake to correlated with pancreatic cancer (101), other research, in contrast, reported that low cholesterol levels result in an increased risk (102).

Overexpression of ACAT1 is related to cholesterol ester accumulation in tumor cells and unwanted cell survival (103); furthermore, the higher absorption of cholesterol by cancer cells than by normal cells is associated with LDLR overexpression (12).

### Ovarian cancer

Contradictory results have appeared regarding blood cholesterol levels and ovarian cancer (104, 105). In one ovarian cancer cell line, high ACAT1 expression was shown, leading to cholesterol ester accumulation. Furthermore, ACAT1 inhibition was reported to suppress cancer cell proliferation (106). Other research documented the importance of HMG-CoA reductase in ovarian cancer, finding that genetically proxied inhibition of HMG-CoA reductase was related to a lower rate of ovarian cancer (107).

### Hematologic malignancies

Studies of the relationship between blood cholesterol levels and leukemia have yielded conflicting results. Lower cholesterol concentrations were reported in patients with chronic lymphocytic leukemia (CLL) and acute lymphocytic leukemia (ALL) than in healthy controls (108, 109), but a different report documented high cholesterol levels in patients with CLL (110). The results of another study demonstrated that in patients with CLL, elevated SREBP2 expression resulted in increased LDLR, thus cholesterol accumulation in the tumor cell cytoplasm was noted as a possible cause of this cancer (111).

## The role of anticholesteremic agents in cancer treatment and patient survival

Cholesterol serves as the component involved in drug resistance in cancer (6). Drug-resistance cells elevate cholesterol uptake and biosynthesis. Increased cholesterol content in cancer cell membrane changes the signaling pathway because of the interaction of cholesterol and different receptors on the surface of these cells. Furthermore, high cholesterol levels convert the entrance drug pathway in malignant cells (112), for example gefitinib-resistant non-small cell lung cancer (NSCLC) cell lines possess higher cholesterol in their lipid raft than gefitinib-sensitive cell lines, as gefitinib exerts a better function on cholesterol-depleted cells (113).

Taken together, surveying a drug that adjusts cholesterol levels for achieving practical and effective treatment.

Statins (HMG-CoA reductase inhibitors) are a group of drugs that can help to lower LDL cholesterol. The advantages of statins for cancer monotherapy or combination therapy have been widely reported. It is suggested that statins reduce cancer cell growth through AKT suppression (114), promote cancer cell apoptosis through Bax overexpression and Bcl2 inhibition (115), and also exert an effect on cell cycle regulatory elements and thus reduce cancer cell proliferation (116).

Numerous studies have documented the beneficial effects of statins on cancer recurrence and mortality (117); for example, lipophilic statins were found to decrease breast cancer recurrence (118).

Other research showed that statin together with palliative care can reduce the mortality rate in patients with hepatocellular carcinoma (119). In addition, statins were found to reduce the mortality rate of colorectal cancer (120, 121). Another study reported beneficial effects of statins on cancer mortality both before and after diagnosis (122). However, statins may have no benefits for patients with advanced cancer (123).

Regarding the interaction of statins with other treatments for cancer, cholesterol-lowering drugs were found to be safe for use along with immunotherapy (124), chemotherapy (125), and radiotherapy (126).

## Conclusion

Cholesterol has a dual effect on the immune response depending on its concentration. High serum levels of cholesterol are related to a higher risk of cancer progression because of their effect on signaling pathways. Tumor cells have complex interactions with their surrounding environment; in generally, tumor cell metabolic pathways are modified by increased cholesterol synthesis and/or uptake. Cholesterol-rich environments change immune cell phenotypes and functions, such that these cells may support cancer cell survival.

Reports of the correlations between blood cholesterol levels and different types of cancer are conflicting. Elevated cholesterol uptake by malignant cells may be associated with low blood cholesterol levels, whereas high blood cholesterol levels in patients with cancer may be viewed as a result of increased cholesterol synthesis in tumor cells. Therefore, cholesterol can potentially act as a cause or an effect of cancer, although the type and stage of the tumor should not be overlooked.

In light of the effects of high cholesterol levels in cancer biology, prescribing cholesterol-lowering drugs may be valuable in cancer therapy. To reduce the side effects of these drugs and achieve better outcomes, targeting the pathways of cancer cell cholesterol metabolism is a potentially helpful avenue for further research.

## Author contributions

Both authors contributed equally to this article and approved the submitted version.

## Acknowledgments

We thank Dr. P. Mokaram, Professor of Biochemistry, for her critical review of this manuscript and K. Shashok (AuthorAID in the Eastern Mediterranean) for improving the use of English.

## Conflict of interest

The authors declare that the research was conducted in the absence of any commercial or financial relationships that could be construed as a potential conflict of interest.

## Publisher’s note

All claims expressed in this article are solely those of the authors and do not necessarily represent those of their affiliated organizations, or those of the publisher, the editors and the reviewers. Any product that may be evaluated in this article, or claim that may be made by its manufacturer, is not guaranteed or endorsed by the publisher.
